# Temporal trends and forecasting of COVID-19 hospitalisations and deaths in Scotland using a national real-time patient-level data platform: a statistical modelling study

**DOI:** 10.1016/S2589-7500(21)00105-9

**Published:** 2021-07-05

**Authors:** Colin R Simpson, Chris Robertson, Eleftheria Vasileiou, Emily Moore, Colin McCowan, Utkarsh Agrawal, Helen R Stagg, Annemarie Docherty, Rachel Mulholland, Josephine L K Murray, Lewis D Ritchie, Jim McMenamin, Aziz Sheikh

**Affiliations:** a School of Health, Wellington Faculty of Health, Victoria University of Wellington, Wellington, New Zealand; b Usher Institute, The University of Edinburgh, Edinburgh, UK; c Department of Mathematics and Statistics, University of Strathclyde, Glasgow, UK; d Public Health Scotland, Glasgow, UK; e HDR UK BREATHE Hub, Edinburgh, UK; f School of Medicine, University of St Andrews, St Andrews, UK; g Centre of Academic Primary Care, University of Aberdeen, Aberdeen, UK

## Abstract

**Background:**

As the COVID-19 pandemic continues, national-level surveillance platforms with real-time individual person-level data are required to monitor and predict the epidemiological and clinical profile of COVID-19 and inform public health policy. We aimed to create a national dataset of patient-level data in Scotland to identify temporal trends and COVID-19 risk factors, and to develop a novel statistical prediction model to forecast COVID-19-related deaths and hospitalisations during the second wave.

**Methods:**

We established a surveillance platform to monitor COVID-19 temporal trends using person-level primary care data (including age, sex, socioeconomic status, urban or rural residence, care home residence, and clinical risk factors) linked to data on SARS-CoV-2 RT-PCR tests, hospitalisations, and deaths for all individuals resident in Scotland who were registered with a general practice on Feb 23, 2020. A Cox proportional hazards model was used to estimate the association between clinical risk groups and time to hospitalisation and death. A survival prediction model derived from data from March 1 to June 23, 2020, was created to forecast hospital admissions and deaths from October to December, 2020. We fitted a generalised additive spline model to daily SARS-CoV-2 cases over the previous 10 weeks and used this to create a 28-day forecast of the number of daily cases. The age and risk group pattern of cases in the previous 3 weeks was then used to select a stratified sample of individuals from our cohort who had not previously tested positive, with future cases in each group sampled from a multinomial distribution. We then used their patient characteristics (including age, sex, comorbidities, and socioeconomic status) to predict their probability of hospitalisation or death.

**Findings:**

Our cohort included 5 384 819 people, representing 98·6% of the entire estimated population residing in Scotland during 2020. Hospitalisation and death among those testing positive for SARS-CoV-2 between March 1 and June 23, 2020, were associated with several patient characteristics, including male sex (hospitalisation hazard ratio [HR] 1·47, 95% CI 1·38–1·57; death HR 1·62, 1·49–1·76) and various comorbidities, with the highest hospitalisation HR found for transplantation (4·53, 1·87–10·98) and the highest death HR for myoneural disease (2·33, 1·46–3·71). For those testing positive, there were decreasing temporal trends in hospitalisation and death rates. The proportion of positive tests among older age groups (>40 years) and those with at-risk comorbidities increased during October, 2020. On Nov 10, 2020, the projected number of hospitalisations for Dec 8, 2020 (28 days later) was 90 per day (95% prediction interval 55–125) and the projected number of deaths was 21 per day (12–29).

**Interpretation:**

The estimated incidence of SARS-CoV-2 infection based on positive tests recorded in this unique data resource has provided forecasts of hospitalisation and death rates for the whole of Scotland. These findings were used by the Scottish Government to inform their response to reduce COVID-19-related morbidity and mortality.

**Funding:**

Medical Research Council, National Institute for Health Research Health Technology Assessment Programme, UK Research and Innovation Industrial Strategy Challenge Fund, Health Data Research UK, Scottish Government Director General Health and Social Care.

## Introduction

Following its emergence in Wuhan, China, in December, 2019, SARS-CoV-2 has spread throughout the world.^1^ In a subset of individuals, SARS-CoV-2 causes a severe form of disease, leading to hospitalisation, admission to intensive care units (ICUs), or death. COVID-19 was declared a pandemic by WHO on March 11, 2020.

A key aspect of the epidemiological response to COVID-19 has been the use of individual patient health-care data—eg, data for those with a positive RT-PCR test for SARS-CoV-2 who have been hospitalised or died.^2^ As the COVID-19 pandemic continues, rich, real-time individual person-level data are needed to better understand the natural population-based dynamics of the disease.^3^ Three different model types—susceptible-exposed-infectious-recovered or susceptible-infectious-recovered models, agent-based models, and curve-fitting models—have been used to project possible numbers of confirmed (new) cases, hospitalisations, and deaths under different scenarios.^4^, ^5^ National-level surveillance platforms can also help to monitor and predict the epidemiological and clinical profile of COVID-19, thus informing public health policy. These platforms have the advantage of including a complete unselected population, in which disease introductions are common and community transmission widespread.^6^ However, most available individual-level data come from specific institutions, highly selected populations, or limited country-wide coverage (eg, limited to one city^3^ or patients with more severe disease^2^).

**Research in context**

**Evidence before this study**

As the COVID-19 pandemic continues, rich, real-time individual person-level data are required to better understand the natural population-based dynamics of the disease. National-level surveillance platforms can help to monitor and predict the epidemiological and clinical profile of the COVID-19 pandemic and thus inform public health policy. These platforms have the advantage of including a complete unselected population, with disease introductions and community transmission. We searched Google Scholar on Sept 10, 2020, for English-language articles relating to national-level surveillance platforms for COVID-19, using the terms “SARS-CoV-2,” “COVID-19,” “surveillance platforms,” “unselected population,” and “individual-level data”. However, in the six studies identified, we found that most available individual-level data could only identify patients from specific institutions, highly selected populations, or with limited country-wide coverage (eg, limited to patients with severe disease or to only one city).

**Added value of this study**

To our knowledge, we have created the first individual patient-level dataset from all primary care, secondary care, mortality, and virological testing data on almost an entire national population. We used this national dataset to investigate the temporal progression of COVID-19 cases, morbidity, and mortality in individuals initially free of the disease across Scotland. Based on these data, we created a novel statistical prediction model for COVID-19 deaths and hospitalisations and used it to accurately forecast the expected impact of the second wave (beginning June 28, 2020) on the Scottish health-care system. COVID-19-related hospitalisations among people testing positive for SARS-CoV-2 within 14 days were associated with being male, and having chronic heart, kidney, or respiratory disease; depression; diabetes; haematological malignancy; hypertension; immunosuppression; pregnancy; splenectomy and anaemia; or transplantation. COVID-19 deaths within 28 days after a positive test were associated with being male or a care home resident, having chronic heart or kidney disease, dementia, haematological malignancy, splenectomy and anaemia, myoneural disease, or having home oxygen. We found decreasing temporal trends in hospitalisation and death rates for those testing positive for SARS-CoV-2 during the second wave. However, we identified an emerging pattern in the second wave of an increasing incidence and proportion of positive SARS-CoV-2 tests in older age groups with multimorbidity.

**Implications of all the available evidence**

These results signalled the need to consider public health interventions to reduce the numbers of hospitalisations and deaths that would otherwise follow. The estimated incidence of infection based on positive tests recorded in this unique data resource yielded accurate forecasts of hospitalisation and death rates for the whole of Scotland. These findings were used by the Scottish Government to inform their response to COVID-19.

We aimed to develop a national dataset of individual patient-level primary and secondary care, mortality, and virological and serological testing data in Scotland to assess the temporal progression of the COVID-19 pandemic, risk factors for COVID-19-related hospitalisation and death, and temporal trends in hospitalisations and deaths by age and risk factor groups. We also aimed to develop a novel statistical prediction model to forecast COVID-19-related deaths and hospitalisations in the second COVID-19 wave in Scotland.

## Methods

### Study design

We linked anonymised individual patient-level primary care, secondary care, mortality, and virological and serological testing data in Scotland and used this national dataset to investigate the temporal progression of COVID-19 in residents of Scotland who had not previously tested positive for SARS-CoV-2. Based on these data, we identified risk factors for COVID-19-related hospitalisation and death and temporal trends in the proportion of hospitalisations and deaths by age and risk factor groups among those with a positive RT-PCR test for SARS-CoV-2. Additionally, we developed a novel 28-day statistical prediction model to forecast COVID-19-related hospitalisations and deaths after a positive test during the second COVID-19 wave in Scotland (ie, from June 28, 2020).

Ethical approval for this study was granted from South East Scotland Research Ethics Committee 02 (12/SS/0201). The Public Benefit and Privacy Panel Committee of Public Health Scotland approved the linkage and analysis of the de-identified datasets for this project (1920-0279).

### Data sources

Almost all residents of Scotland (including children) are registered with primary care, which provides a comprehensive array of health-care services that are free at the point of care, including the issuing of prescriptions for medications. Access to secondary care is typically through a general practitioner (GP) based within a primary care practice or via the emergency department or out-of-hours primary care services. Additionally, during the acute phase of the pandemic (ie, from March 23, 2020), community-based COVID-19 hubs (GP-led services designed to separate patients with COVID-19 and reduce the risk of nosocomial infections) were established.

We used data from all 940 Scottish primary care practices.^7^ Clinical data collected by primary care in Scotland have consistently been shown to be of high quality (90% completeness and accuracy^8^) and their value for epidemiological research has been repeatedly demonstrated.^9^, ^10^, ^11^, ^12^ These data were linked to three other national datasets: the Electronic Communication of Surveillance in Scotland (national database for all virology testing), the Scottish Morbidity Record (which records hospitalisations, including ICU stay), and National Records Scotland (for death certification; appendix p 1).^13^

Our cohort included all patients of any age registered with any primary care practice in Scotland on Feb 23, 2020, who were followed up from March 1, 2020, to Nov 8, 2020.

### Risk factors and outcomes

We assessed several factors potentially associated with COVID-19 morbidity and mortality and included them in our prediction model: sex, age, socioeconomic status, urban or rural residence, and risk groups based on clinical characteristics, all of which were identified from primary care records. All participants were included, regardless of data availability; participants with missing socioeconomic status were included as a separate category. Socioeconomic status was determined on the basis of the Scottish Index of Multiple Deprivation (SIMD). The SIMD classification is based on deprivation quintiles: quintile 1 refers to the most deprived and quintile 5 refers to the most affluent. SIMD was assigned according to residential postcode. Urban or rural residence was classified using the urban rural six-fold classification (UR6), which is the definition used to determine rurality in Scotland: 1 is assigned to large urban areas (ie, ≥125 000 residents) and 6 is assigned to remote rural areas (ie, <3000 residents and >30 min drive to urban areas).^14^ Clinical risk groups were defined using the primary care clinical Read coding system, with codes grouped into 17 categories: care home residence, chronic heart disease, chronic kidney disease, chronic liver disease, chronic respiratory disease, dementia, depression, diabetes, haematological malignancy, home oxygen, hypertension, immunosuppression, multiple sclerosis and degerenative disease, myoneural disease, splenectomy and anaemia, transplantation, and pregnancy (appendix pp 2–3). The included conditions were considered to be potential COVID-19 risk factors based on previous studies focusing on severe disease from influenza virus and from early epidemiological data.^15^, ^16^ Patients were classified as members of a clinical risk group if there was a record of a Read code associated with the relevant diagnosis in the patient's primary care record before Feb 23, 2020.

For COVID-19-related hospitalisations, we included individuals who were hospitalised within 14 days following a positive RT-PCR test for SARS-CoV-2, including those who tested positive while hospitalised, or those who were hospitalised with an admission diagnosis of COVID-19. COVID-19-related deaths were all-cause deaths occurring within 28 days after a positive test for SARS-CoV-2 that were registered with National Records Scotland and included death certification, or deaths with COVID-19 on the death certificate as the cause of death.

### Forecasting model and statistical analysis

Our novel survival prediction model is based on clinical and demographic characteristics of individuals with a positive RT-PCR test for SARS-CoV-2, in addition to age; sex; UR6; number of comorbidities (ie, risk groups), including care home residency; and socioeconomic status. The prediction model is derived from data from March 1 to June 23, 2020 (the first wave of the COVID-19 pandemic in Scotland). Clinical risk group information is based on the clinical characteristics of the cohort on March 1, 2020, and used to calculate the number of comorbidities each individual had.

For those who tested positive for SARS-CoV-2 in Scotland during the first wave, a Cox proportional hazards model was used to estimate the association between each of the clinical risk groups and time to hospitalisation or death from the date the person had a positive test result. Risk factors informative to the model were used; these factors were then used to calculate the count of comorbidities that was included in the prediction model. Demographic data (age, sex, socioeconomic status, and UR6) were also included in the prediction model. Age (in years) and time (measured in days from March 1, 2020) were included in the model as penalised splines. Backwards selection of the risk group variables was used to obtain a parsimonious model and five-fold cross-validation to estimate concordance.

Forecasts from June 24 to Nov 8, 2020, were produced each week based upon those testing positive in this period. For each positive case, using their risk profile (eg, age, sex, and number of comorbidities), we calculated individual risk of becoming hospitalised (within 14 days after a positive test) or death (within 28 days after a positive test), on the basis of the identical characteristics of patients who previously tested positive for SARS-CoV-2 and their outcomes. The last forecast we present is for 28 days after Nov 10, 2020.

We did not forecast the temporal effect (time to hospitalisation or death following a positive test) using the spline term, but instead used the latest values of the temporal effect from the observed period (ie, June, 2020). Each week, we fitted a generalised additive spline model to the daily SARS-CoV-2-positive cases over the previous 10 weeks and used this to forecast the total number of cases per day over the following 4 weeks.^17^ The age and risk group pattern of cases in the previous three weeks was then used to select a stratified sample of individuals from our cohort who had not previously tested positive (eg, data from Oct 19 to Nov 8, 2020, were used for 28-day predictions starting from Nov 10). Future cases in each group were then sampled from a multinomial distribution, with the total cases coming from the spline growth model. For example, if we forecasted 1000 cases the next day and 5% of cases in the previous week were aged 65 years or older and had two comorbid conditions, we then sampled 50 people who had these characteristics out of the total previously uninfected population and used their patient characteristics (including age, sex, comorbidities, socioeconomic status, and UR6) values to predict their probability of being hospitalised or dying. In this way, the clinical characteristics of future cases were used to forecast hospital admissions and deaths.

Forecasts are based on extrapolation of the cases testing positive and scenarios (eg, non-pharmacological public health interventions to suppress transmission) that assume a halving in the growth of positive cases and a reduction to a growth rate of zero. We also produced a set of concordance values for the model (with values >0·80 indicating very good concordance), fitting a period up to June 23, 2020, and then for two test periods representing different stages of the second wave. During the first test period (June 24 to Sept 30, 2020), there were low numbers of COVID-19 hospitalisations and deaths. The second period (Oct 1 to Nov 8, 2020) represents a time when hospitalisations and deaths were much higher.

Predictions were sampled from a probability distribution, with predictions run 20 times to allow calculation of 95% prediction intervals (PIs).

Analyses were done in R (version 3.6.1). All code developed for this forecasting method has been included in our GitHub area.

### Role of the funding source

The funders of the study had no role in study design, data collection, data analysis, data interpretation, or writing of the report.

## Results

Our cohort included 5 384 819 people registered with a GP on Feb 23, 2020, representing 98·6% of the entire estimated population residing in Scotland during 2020 (estimated at 5 463 300 from the 2018 census).

Based on data from March 1 to June 23, 2020, COVID-19-related hospitalisations and deaths were associated with several patient characteristics including male sex (hazard ratio [HR] for hospitalisation 1·47, 95% CI 1·38–1·57; HR for death 1·16, 1·49–1·76). Among comorbidities, the highest HR for hospitalisation was found for transplantation (4·53, 1·87–10·98) and the highest HR for death was found for myoneural disease (2·33, 1·46–3·71; appendix pp 4–5). COVID-19-related hospitalisations were also associated with having chronic heart, kidney, or respiratory disease; depression; diabetes; haematological malignancy; hypertension; immunosuppression; pregnancy; and splenectomy or anaemia (appendix p 4). COVID-19 death was also associated with being a care home resident, having chronic heart or kidney disease, dementia, having a haematological malignancy, splenectomy or anaemia, or having home oxygen (appendix p 5). Decreased risk of COVID-19-related hospitalisation and mortality was associated with affluent socioeconomic status (SIMD quintile 5; appendix pp 4–5).

774 491 people who were tested for SARS-CoV-2 using RT-PCR over the study period were linked into the study population, representing 14·4% of the cohort (appendix p 7). 223 866 people were tested in the period up to June 23, 2020, with a further 526 591 newly tested between June 24 and Nov 8, 2020. The number of tests per week increased during the study period (appendix p 8).

**Figure 1:**
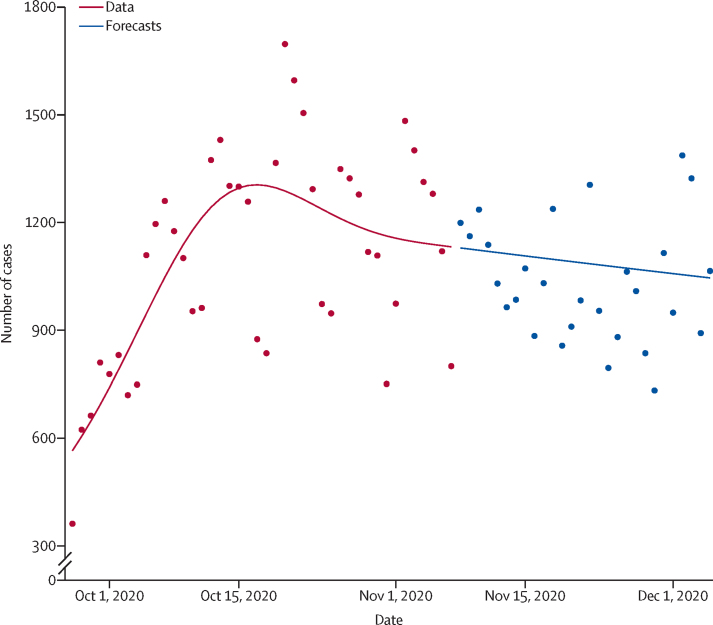
Extrapolation of cases testing positive for SARS-CoV-2 Figure shows testing data up to Nov 8, 2020, with extrapolation from Nov 10 to Dec 8.

SARS-CoV-2 was confirmed in 75 173 (9·7%) of the 774 491 Scottish residents tested using RT-PCR up to Nov 8, 2020, with extrapolation of positive cases up to December shown in figure 1. There is evidence of a slowing down or turning point in the daily number of SARS-CoV-2 cases around Oct 17, 2020, probably due to restrictions imposed on household visits and hospitality on Sept 23, followed by further restrictions on hospitality on Oct 9. Dynamics in test positivity varied over time by sex, age, risk group, and socioeconomic status. During the initial phase of the pandemic, the proportion of positive tests among female individuals was around 25% (*vs* 75% among male individuals); this percentage increased substantially to more than 65% by June, 2020, dropped during July and August to less than 50%, and finally increased to 55% by October, 2020 (appendix p 9). Up to June 23, 2020, 25% of those testing positive were aged 80 years or older. The percentage of young adults (aged 18–39 years) testing positive rose from 25% in April to 50% in August, 2020; this percentage declined from September, 2020, onwards, with concomitant increases among those older than 40 years of age (appendix p 10). During the first wave, 10% of those testing positive had five or more clinical conditions deemed to be COVID-19 risk factors; this percentage dropped to around 2% by August, 2020, followed by an increase to 15% by October. There was a steady rise in the percentage of those testing positive who did not have any relevant clinical conditions to a peak of more than 70% of cases in August, followed by a decline to 50% in October, 2020 (appendix p 11). 30% of people testing positive at the beginning of the pandemic were among the most affluent (SIMD quintile 5); this proportion declined to 10% by July, 2020, and then increased slightly to 18% by October, 2020. Overall, a higher proportion in large and other urban areas tested positive during 2020 than in remote small towns and remote rural areas (appendix p 11). No discernible changing temporal patterns were identified for urban versus rural areas.

5104 (6·8%) of 75 173 people who tested positive for SARS-CoV-2 were hospitalised for COVID-19 up to Nov 8, 2020. Of the 5 384 819 people in the cohort, 3796 died of COVID-19. The COVID-19 death rate in Scotland during the studied period was 703 deaths per 1 million population.

**Figure 2:**
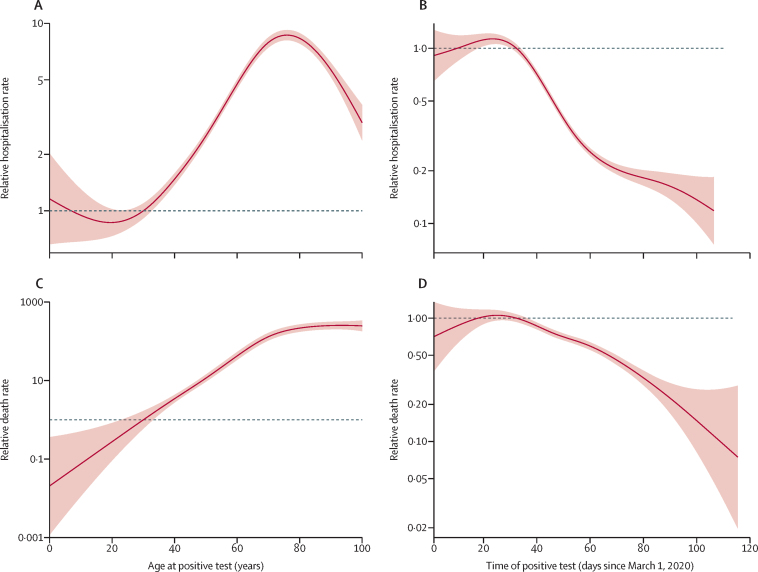
COVID-19-related hospitalisations and deaths by age and time trends (A) Hospitalisation rate by age during the first wave (March 1 to June 23, 2020), relative to 30 years of age. (B) Hospitalisation rate over time during the first wave, relative to day 31 (April 1, 2020). (C) Death rate by age during the first wave, relative to 30 years of age. (D) Death rate over time during the first wave, relative to day 31 (April 1, 2020). Vertical axes for graphs C and D are on a log scale.

During the first wave (up to June 23, 2020), relative hospitalisation and death rates among individuals testing positive for SARS-CoV-2 using RT-PCR decreased over time (figure 2B, D). Hospitalisation rates increased with age, peaking at around 75 years of age with a decline thereafter (figure 2A). Deaths also increased with age, plateauing at around 80 years of age (figure 2C).

**Figure 3:**
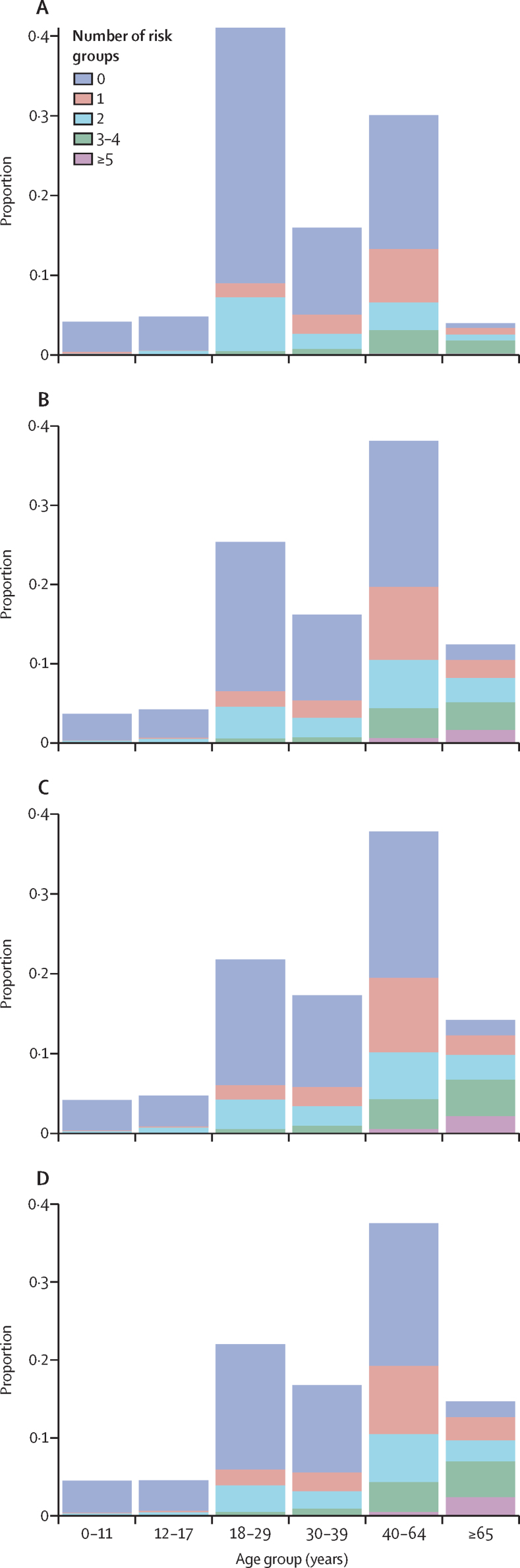
Distribution of RT-PCR-confirmed SARS-CoV-2 cases by age and risk groups in August, October, and November, 2020 Data shown in panels C and D were used in the forecast, with panels Aand B illustrating the distribution at earlier timepoints. (A) About 150 positive cases per day were recorded in the week beginning Aug 29, 2020 (131 202 total tests). (B) About 1000 positive cases per day were recorded in the week beginning Oct 10, 2020 (117 920 total tests). (C) About 1400 positive cases per day were recorded in the week beginning Oct 17, 2020 (126 972 total tests). (D) About 1000 positive cases per day were recorded in the week beginning Nov 1, 2020 (124 815 total tests).

The age and risk pattern of the 25 827 RT-PCR-positive cases in the last 3 weeks of the study is shown in the table, presented as breakdown of proportions by age and number of risk groups; the age and risk pattern for all tests in the previous 3 weeks can be found in the appendix (p 6). The proportion of older people and people with comorbidities testing positive for SARS-CoV-2 increased from the end of the first test period (Aug 29) to Oct 24, 2020 (figure 3; appendix pp 10–11).

**Table:** Proportion of cases by age and risk factors in the last 3 weeks of the study (Oct 19 to Nov 8, 2020)

	**Number of risk groups**					**All risk groups**
	0	1	2	3–4	≥5	
0–11 years	0·0315	0·000852	0·00426	0	0	0·0366
12–17 years	0·0456	0·00213	0·00596	0	0	0·0537
18–29 years	0·304	0·0266	0·0614	0·00565	0·000807	0·398
30–39 years	0·0842	0·0168	0·0158	0·00368	0	0·120
40–64 years	0·144	0·0729	0·0493	0·0351	0·00420	0·306
≥65 years	0·0119	0·0212	0·0195	0·0239	0·00885	0·0854
All ages	0·621	0·140	0·156	0·0683	0·0139	1·00

This age and risk group pattern was used to select a stratified sample of individuals who had not previously tested positive from the cohort. Cases were sampled from a multinomial distribution and total cases came from the spline growth forecasting model.

The concordance value for hospitalisations over the period March 1 to June 23, 2020, was 0·84. For the two test periods (June 24 to Sept 30, 2020, and Oct 1 to Nov 8, 2020), the concordance for hospitalisations was 0·80 for each period. The concordance value for deaths was 0·84 in the period from March 1 to June 23, 0·93 for the first test period (June 24 to Sept 30), and 0·92 for the second test period (Oct 1 to Nov 8).

**Figure 4:**
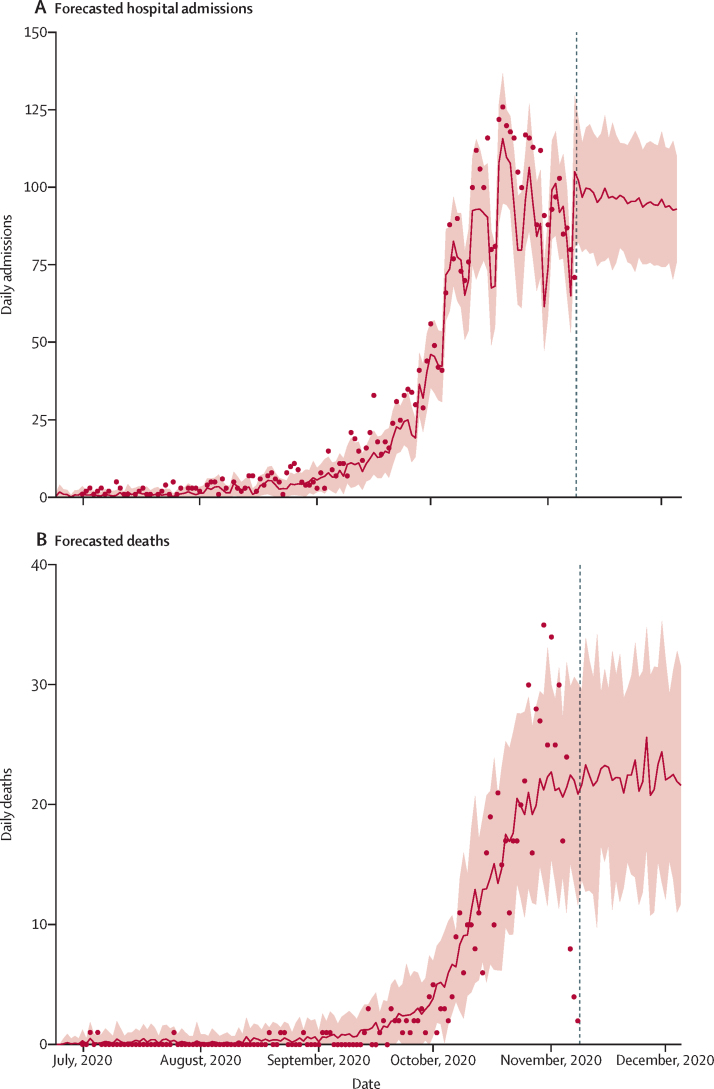
Forecasted hospitalisations and deaths (from Nov 10, 2020) among those who tested positive for SARS-CoV-2 Hospital admissions (A) and deaths (B) among people who tested positive for SARS-CoV-2 per day. The red line shows mean predictions, with 95% prediction intervals. The model is fitted to data up to June 23, 2020, and the predictions from July 1, 2020, to Nov 8 are based on the clinical characteristics of the tested positive cases. The predictions from Nov 10 (the dotted line) onwards are based on forecasted cases. The saw-tooth pattern in the hospitalisation predictions in September and October is due to the weekend pattern of testing, which led to a relatively short time between patients testing positive and being hospitalised. This is averaged out in the projections into the future.

By Oct 15, 2020, there were about 70 hospitalisations for COVID-19 and ten COVID-19-related deaths per day in Scotland. On Nov 10 (28 days later) hospitalisations on Dec 8, 2020, were forecast to increase to 90 per day (95% PI 55–125) and deaths to 21 per day (12–29; figure 4). These forecasts assumed no changes in the growth of the epidemic or in the demographic and clinical characteristics of patients testing positive after Nov 10.

As an illustration of the changing dynamics, forecasts made on Oct 24 for hospitalisations and deaths up to Nov 21, 2020, are presented for different SARS-CoV-2 growth rates (appendix pp 13–15). The original forecasts showed increasing hospitalisations and deaths, reaching 290 hospitalisations (95% PI 260–320) and 30 deaths (20–55) per day by mid-November (appendix p 13). If the growth rate of SARS-CoV-2 was halved, the forecast at the same timepoint became 220 hospitalisations per day (190–250) and 25 deaths per day (16–34; appendix p 14). If the growth rate of SARS-CoV-2 was reduced to zero, the forecast at the same timepoint showed hospitalisations levelling off at 155 per day (130–175) and deaths at 18 per day (10–27; appendix p 15). Zero growth reflects the situation on Nov 11, 2020, following the introduction of movement restrictions in Scotland during the mid-October school break and continued restrictions for most of central Scotland.

## Discussion

In this study, a whole-population dataset with individual patient-level data was created and used to analyse the temporal patterns of the COVID-19 pandemic in Scotland in 2020. We found that morbidity and mortality occurred disproportionately in the most socioeconomically deprived and in those with the most clinical risk factors. Numbers of positive RT-PCR tests increased rapidly and peaked among women and in the oldest age groups both before and immediately after the first national lockdown (March 23 to May 29, 2020). During the initial wave, up to June, 2020, COVID-19 hospitalisation and mortality rates decreased for those testing positive, with mortality being particularly high for the oldest (≥80 years) residents of Scotland. In the second wave, groups with lower levels of SARS-CoV-2 infection during the first wave (younger, male, and affluent groups) saw increases in infection rates

The observed number of COVID-19-related hospitalisations on Dec 8, 2020 (n=104), was 14 higher than our forecasted prediction of 90, and was within our 95% PI (55–125).^18^ The observed number of deaths on the same day (n=27) was six deaths higher than our forecast, and was also within our 95% PI (12–29).

A substantial strength of this study was our ability to describe whole-population data, which contrasts with previous studies that have, at best, been able to study only subsamples of the national population.^19^, ^20^ Our whole-population dataset includes patients registered with all general practices in Scotland.^13^ Almost all health care in Scotland is provided via the National Health Service (NHS), with almost all people resident in Scotland registered with a GP. Each person in Scotland has a unique identifier that is used in every health contact with the NHS. This Community Health Index^21^ number allows for highly accurate data linkage at an individual patient level to national, routinely collected data sources. These results are likely to have excellent generalisability across the UK and reasonable generalisability to countries with similar health systems, population structures (predominant ethnicity 90% White, with a population density of 67·2 people per km^2^), and experiences with the pandemic. Our rapid access to this data source through the NHS National Institute for Health Research Pandemic Preparedness Portfolio enabled us to quickly enhance a pandemic influenza platform (EAVE I) to provide granular real-time data on the COVID-19 pandemic.^22^, ^23^

This dataset has the advantage of being populated entirely by routinely collected data, and consequently can be used for clinical practice. However, given the observational nature of the study, there is potential for confounding, due to insufficiently granular, or a lack of, measurement of variables. A small proportion of people resident in Scotland might not be registered in primary care, including the homeless (those without permanent abode) and travelling people. Although the prediction models use up-to-date data, they are still sensitive to the fitted temporal trend. This temporal trend was most imprecise at the end of the observed data series (June 23, 2020), and these values were used in the prediction. The forecasts assume that recent trends will persist and that no further local restrictions are implemented (eg, in response to a more susceptible case mix of people being infected). The predictions also assumed no change in the relationship between clinical and demographic characteristics and outcomes. Furthermore, change in accessibility of testing over time could result in differential misclassification, altering the case mix of those being tested. During the second wave, there was a move towards proactive case finding, early warning in key sectors including schools, and increased testing, leading to identification of more asymptomatic patients.^24^ In particular, the changing test access criteria make it difficult to interpret differences in time from positive test to hospitalisation and death during the first and second waves. Prolongation of the interval between detection of infection and death (an estimated 17·8 days between onset and death^17^) might have resulted in underestimation of deaths forecast in the short term (eg, 0–20 days). Bias might have also occurred due to differences in health-care-seeking behaviours between patients; in particular, health-care workers might have been less likely to seek formal medical advice or assessment, which might have decreased the effect size among this group.^25^ Unmeasured confounders might also have influenced our estimates. The statistical model approach adopted in our analysis has advantages and disadvantages compared with a dynamic transmission model. The advantage of our statistical model approach is that it is likely to be more parsimonious and it is not necessary to understand why the interventions worked to fit our models. A disadvantage is that further refinements will be needed to improve the model as more data become available—eg, transmission modelling and who-acquires-infection-from-whom matrices.^26^

Given the early and wide-ranging lockdown measures imposed on all people resident in Scotland from early 2020, the initial impact of COVID-19 on the Scottish population was substantial. Despite high adherence to lockdown measures,^27^ multiple introductions of infection, particularly from Europe, caused an accelerated outbreak.^6^ Increasing proportions of at-risk people (those with older age and more at-risk comorbidities) testing positive for SARS-CoV-2 resulted in substantial morbidity and mortality among these groups. Various measures, such as test, trace, and isolate; shielding;^28^ continually updated evidence-based guidance for care homes; and prioritised personal protective equipment, have nonetheless resulted in these groups being better protected in subsequent pandemic waves. Emerging patterns in the second wave included an increasing number of test-positive cases in late August for all age groups younger than 40 years. This is likely to have been due to the opening of schools on Aug 11, 2020, resulting in increased household transmission in Scotland. A pattern of increasing incidence and proportion of positive tests in older age groups with multimorbidity was also of concern, signalling the need to consider public health interventions to reduce the number of hospitalisations and deaths that might have otherwise followed. Together with other public health surveillance data, policy makers were able to use these data to inform decisions on when physical distancing measures should be introduced.^29^ This novel forecasting method is also providing an indication of the relative success (or otherwise) of public health measures at a population level.

To our knowledge, this is the first national dataset to contain near real-time patient-level data for the whole population. The linkage of multiple data sources, including the rich data available from primary care and all SARS-CoV-2 laboratory testing, hospitalisations, and deaths, means that accurate and timely forecasting can be carried out and used to inform government policy.

## Data sharing

All code used in this study is publicly available online. The data used in this study are sensitive due to individual patient-level data and will not be made publicly available.

## Declaration of interests

AS is a member of the Scottish Government Chief Medical Officer's COVID-19 Advisory Group and the New and Emerging Respiratory Virus Threats Risk Stratification Subgroup. CRS declares funding from the Medical Research Council (MRC), National Institute for Health Research (NIHR), Chief Scientist Office, and New Zealand Ministry for Business, Innovation and Employment and Health Research Council, during the conduct of this study. CR reports grants from the MRC and Public Health Scotland, during the conduct of the study, and is a member of the Scottish Government Chief Medical Officer's COVID-19 Advisory Group, Scientific Pandemic Influenza Group on Modelling, Medicines and Healthcare products Regulatory Agency, Vaccine Benefit and Risk Working Group. JM reports grants from EAVE II funded by MRC/NIHR/Scottish Government and grants from IMOVE-COVID-19 funded by WHO (Europe) and the European Centre for Disease Prevention and Control, during the conduct of the study. HRS reports that her institution received grants from the MRC and UK Research and Innovation, during the conduct of the study, and that she received personal fees from Scottish Parliament, outside the submitted work. JLKM reports being the clinical lead of Health Protection for NHS Fife and a member of the COVID-19 National Incident Management Team. All other authors declare no competing interests.
