# Supplementary material for: Temporal trends and forecasting of COVID-19 hospitalisations and deaths in Scotland using a national real-time patient-level data platform: a statistical modelling study
Source: Lancet Digit Health. 2021 Jul 5;3(8):e517–25. doi: 10.1016/S2589-7500(21)00105-9 (PMC8257056; doi:10.1016/S2589-7500(21)00105-9)
Supplement: Supplementary appendix [file mmc1.pdf]

THE LANCET
Digital Health

Supplementary appendix

This appendix formed part of the original submission and has been peer reviewed. We post it as supplied by the authors.

Supplement to: Simpson CR, Robertson C, Vasileiou E, et al. Temporal trends and forecasting of COVID-19 hospitalisations and deaths in Scotland using a national real-time patient-level data platform: a statistical modelling study. *Lancet Digit Health* 2021; published online July 5. https://doi.org/10.1016/S2589-7500(21)00105-9.

Supplemental Material

Contents

Supplemental Table 1 Data sources 1
Supplemental Table 2 Variable Descriptors 2
Supplemental Table 3 Hazard ratios and 95% confidence intervals for COVID-19 hospitalisations March to June 2020 4
Supplemental Table 4 Hazard ratios and 95% confidence intervals for COVID-19 deaths March to June 2020 5
Supplemental Table 5 The age and risk group pattern of tested individuals from primary care risk groups in the last three weeks of the study 6
Supplemental Figure 1 Study flow diagram – data from 1 March to 8 November 2020 7
Supplementary Figure 2 Weekly total tests performed throughout the study period 8
Supplemental Figure 3: RT-PCR confirmed SARS-CoV-2 cases over time for females 9
Supplemental Figure 4: RT-PCR confirmed SARS-CoV-2 cases over time by age band 10
Supplemental Figure 5 RT-PCR confirmed SARS-CoV-2 cases over time for risk groups (co-morbidities) and Urban/Rural settings 11
Supplemental Figure 6 RT-PCR confirmed SARS-CoV-2 cases over time for socioeconomic status quintiles 12
Supplemental Figure 7 Hospitalisation and death forecasts on October 24th 2020 13
Supplemental Figure 8 Hospitalisation and death forecasts on October 24th 2020 assuming that the growth rate is halved 14
Supplemental Figure 9 Hospitalisation and death forecasts on October 24th 2020 assuming that the growth rate is zero 15
References 16

Supplemental Table 1 Data sources

Data source	Data description and criteria for inclusion	Period of data covered
Laboratory data	The Electronic Communication of Surveillance in Scotland (ECOSS) system of Public Health Scotland is a database that holds surveillance data on various microorganisms (e.g. influenza virus and coronavirus) and infections reported from diagnostics and reference laboratories (including NHS and UK Government testing centre data).[1] Data on laboratory results for all reverse transcriptase PCR (RT-PCR) tests carried out in Scotland are collated by ECOSS.	1 March 2020 to 8 November 2020
Primary care	Data from all patients registered in general practices. Previous observational studies have shown over 91% completeness of capture of contacts and accuracy of clinical event coding (Read codes) among practices in Scotland.[2] Clinical at-risk groups - certain underlying medical conditions where people are at-risk of COVID-19 related complications will be calculated from baseline and include a look back of 10 years to determine whether a person had a co-morbidity Read code. Primary care data includes general practice prescribing.	1 January 2000 to 8 November 2020
Secondary care	The Scottish Morbidity Record (SMR) database will be used to derive information for all in-patient hospitalisations and emergency admissions in Scotland, which is maintained by the Information Services Division. Regular validation checks are applied to the SMR database. The latest data quality assessment of these SMR datasets have shown over 90% completeness and accuracy in consistency with previous years.[3]	1 March to 8 November 2020
National Records Scotland	The National Records of Scotland (NRS), Death certificate states COVID-19 was relevant to the cause of death and death certificate states ‘presumed’ or ‘suspected’ COVID-19 was relevant to cause of death. This includes people who died 28 days post-positive SARS-CoV-2 RT-PCR test.	1 March to 8 November 2020

Supplemental Table 2 Variable Descriptors

Data source	Description
Scottish Index of Multiple Deprivation (SIMD)	The SIMD classification is based on deprivation quintiles. Quintile 1 refers to the most deprived and quintile 5 refers to the least deprived. The SIMD is a combination of 38 indicators of the following seven domains: income, employment, health, education, housing, geographical access to services and crime based on postcode – providing a measure of relative deprivation at the data zone level (between 500 and 1,000 household residents).
Urban Rural Location	The urban/rural location will be determined based on the urban/rural six-fold classification (UR6). The UR6 is the definition of rural areas in Scotland: 1 is assigned to large urban areas and 8 is assigned to remote rural areas based on postcode.
**Risk factors (Read Codes):**	
Care home residence	'9N1G.','13F61','13FT.','13FV.','13FX.','13FK.','13F5.','13F52','13F7.','13F71','13F72','13F6.'
Chronic respiratory disease	'A115.','H3%','C370.','H40%','H41%','H42%','H43%','H44%','H45%','H46..','H460.','H460z','H464%','H46z% H47y0','H48% H4y%','H4z%','H5410.','H55%','H563%','H57%','H582.','H583.','H591.','H592.','H593.','Hyu3%','Hyu40','Hyu41','Hyu48','Hyu5%','Q3170'
Chronic heart disease	'33BA.','G1%','G21%','G220.','G222.','G23%','G3%','G41%','G54%','G55%','G58%','G5y1.','G5y3%','G5y4%','G5y6.','G5y7.','G5y8.','G5yy2','G5yy6','G5yy9','G5yyA','Gyu1%','Gyu3%','Gyu4%','Gyu55','Gyu56','Gyu57','Gyu58','Gyu59','Gyu5A','Gyu5B','Gyu5C','Gyu5D','Gyu5M','Gyu5N','Gyu5P','Gyu5Q','Gyu5R','Gyu5S','Gyu5T','P5%','P60%','P61%','P62%','P63%','P64%','P65%','P66%','P67%','P68%','P6W%','P6X%','P6y..','P6y0%','P6y1%','P6y2%','P6y3%','P6y63','P6y64','P6y6z','P6yy%','P6z..','P6z2.','P6z3.'
Chronic kidney disease	'1Z12.','1Z13.','1Z13.1Z15','1Z16.','1Z1B.','1Z1C.','1Z1D.','1Z1E.','1Z1F.','1Z1G.','1Z1H.','1Z1J.1Z1K.','1Z1L.','K01%','K02%','K0320','K0325','K0A3%','K05%','K0D..','K0E..','Kyu21','7B00%','7B012','7B015','7B063','8L50.','SP083','TB001','ZV420'
Chronic liver disease	'A707%','J6...','J61%','J62y.','J62z.','J6353','J6354','J6355','J6356','J63B.','PB61%','PB63%','PB6y1','J623.','J624.','J625.','J63A.','J6617','Jyu71','SP143'
Immunosuppression	'PK01.','PK06.','G74y6','14N7.','7840%','D4154','D4156','2J30.','2J31.','A788%','A789%','43C3.','AyuC%','C332.','C332z','C333%','D41y1'
Transplantation	'7450%','7800%','7830%','7900%','7901%','8C31.'
Splenectomy/Anaemia	'R0920','2C5%','D21%'
Haematological malignancies	'B6%'
Multiple Sclerosis and degenerative disease	F20%','F21%','F22%','F23%','F24..','F240%','F241%','F242.','F24y%','F24z.','F2A..','F2Az.','Fyu9%','G669.', 'F1...','F10%','F11%','F12%','F13..','F130%','F1322','F134.','F135.','F1350','F135z','F136%','F137.','F1370','F1371','F137y','F137z','F13A.','F13X.','F14%','F15%','F16..','F160%','F161%','F162.','F163.','F1631','F163z','F16y%','F16z.','F17..','F174.','F1y..','F1z..'
Myoneural Disease	'D41y1','D41y2','F38%','Fyu8.','Fyu80','Fyu83','Fyu84','Fyu85'
Diabetes	'C10%.','Cyu2%','L1805','L1806','L1807','L180X','Lyu29.'

Pregnancy	'62%','L02..','L03..','L04..','L20..','Ly0..','7F19.','7F150','7F14.','L395.','7F16.','L396.','7F17.','L3983','7F12.','L3984','7F133','L264.','7E086'
Home oxygen	'6639.','663E.','66Yj.','66Yk.','66Yl.','745E%','7L1Q%','877%'
Dementia	'E00%','E012.','E02y1','E041.','Eu00%','Eu01%','Eu02%','Eu03%','Eu04%'
Hypertension	'G2%'
Depression	'E11%','E130.','E135.','E2003','E204.','E2112','E290%','E291%','E2B%'

Supplemental Table 3 Hazard ratios and 95% confidence intervals for COVID-19 hospitalisations March to June 2020

Patient Risk Group*	Total n	% Total	Adjusted Hazards Ratio	Lower 95% Confidence Limit	Upper 95% Confidence Limit
Sex: Female†	2784106	51.19	1.00	-	-
Male	2654924	48.81	1.47	1.38	1.57
SIMD Quintile 1†	1100475	20.23	1.00	-	-
SIMD Quintile 2	1068771	19.65	0.88	0.81	0.96
SIMD Quintile 3	1056918	19.43	0.79	0.71	0.86
SIMD Quintile 4	1076820	19.80	0.75	0.68	0.82
SIMD Quintile 5	1084255	19.93	0.70	0.63	0.77
SIMD Quintile Unknown	51791	0.95	0.93	0.71	1.22
Care Home Resident	22229	0.41	0.42	0.37	0.48
Chronic Heart Disease	316115	5.81	1.14	1.06	1.24
Chronic Kidney Disease	170176	3.13	1.16	1.06	1.27
Chronic Liver Disease	89966	1.65	1.13	0.97	1.31
Chronic Respiratory Disease	766820	14.10	1.24	1.15	1.33
Dementia	35598	0.65	0.62	0.55	0.70
Depression	545417	10.03	1.10	1.02	1.20
Diabetes	292956	5.39	1.30	1.20	1.40
Haematological Malignancy	19378	0.36	1.40	1.11	1.75
Hypertension	753869	13.86	1.11	1.03	1.19
Immunosuppression	32009	0.59	1.60	1.25	2.05
Pregnancy	57908	1.06	2.48	1.63	3.76
Splenectomy and Anaemia	62638	1.15	1.16	1.00	1.35
Transplant	1142	0.02	4.53	1.87	10.98

* Includes only coefficients informative to the model
† Reference groups – Female and most deprived socioeconomic status

Supplemental Table 4 Hazard ratios and 95% confidence intervals for COVID-19 deaths March to June 2020

Patient Risk Group*	Total n	% Total	Adjusted Hazards Ratio	Lower 95% Confidence Limit	Upper 95% Confidence Limit
Sex: Female†	2784106	51.19	1.00	NA	NA
Male	2654924	48.81	1.62	1.49	1.76
SIMD Quintile 1†	1100475	20.23	1.00	NA	NA
SIMD Quintile 2 †	1068771	19.65	0.98	0.87	1.10
SIMD Quintile 3	1056918	19.43	0.96	0.85	1.09
SIMD Quintile 4	1076820	19.80	0.92	0.81	1.04
SIMD Quintile 5	1084255	19.93	0.77	0.68	0.88
SIMD Quintile Unknown	51791	0.95	1.22	0.94	1.58
Care Home Resident	22229	0.41	1.26	1.13	1.39
Chronic Heart Disease	316115	5.81	1.12	1.02	1.22
Chronic Kidney Disease	170176	3.13	1.17	1.07	1.29
Chronic Respiratory Disease	766820	14.10	1.09	0.99	1.20
Dementia	35598	0.65	1.16	1.06	1.28
Haematologic Malignancy	19378	0.36	1.37	1.03	1.83
Home Oxygen	4641	0.09	1.62	1.14	2.30
MS and Degenerative Diseases	72774	1.34	1.12	0.99	1.25
Myoneural Disease	3272	0.06	2.33	1.46	3.71
Splenectomy and Anaemia	62638	1.15	1.21	1.04	1.42

MS: Multiple Sclerosis
***** Includes only coefficients informative to the model
† Reference groups – Female and most deprived socioeconomic status

Supplemental Table 5 The age and risk group pattern of tested individuals from primary care risk groups in the last three weeks of the study

	Number of Risk Groups*					
**Age Group**	**0**	**1**	**2**	**3-4**	**5+**	**Total in age group**
0-11	0.096559	0.002071	0.005213	0.000258	0	0.104100
12-17	0.050129	0.002524	0.007572	0.000546	0	0.060771
18-29	0.117482	0.016122	0.026486	0.005481	0.000072	0.165643
30-39	0.096260	0.025023	0.022685	0.008303	0.000227	0.152498
40-64	0.160935	0.086093	0.055630	0.039291	0.005851	0.347801
65+	0.027197	0.035778	0.036139	0.050252	0.019821	0.169187
All ages total	0.548563	0.167611	0.153724	0.104131	0.025971	1.000000

Supplemental Figure 1 Study flow diagram – data from 1 March to 8 November 2020

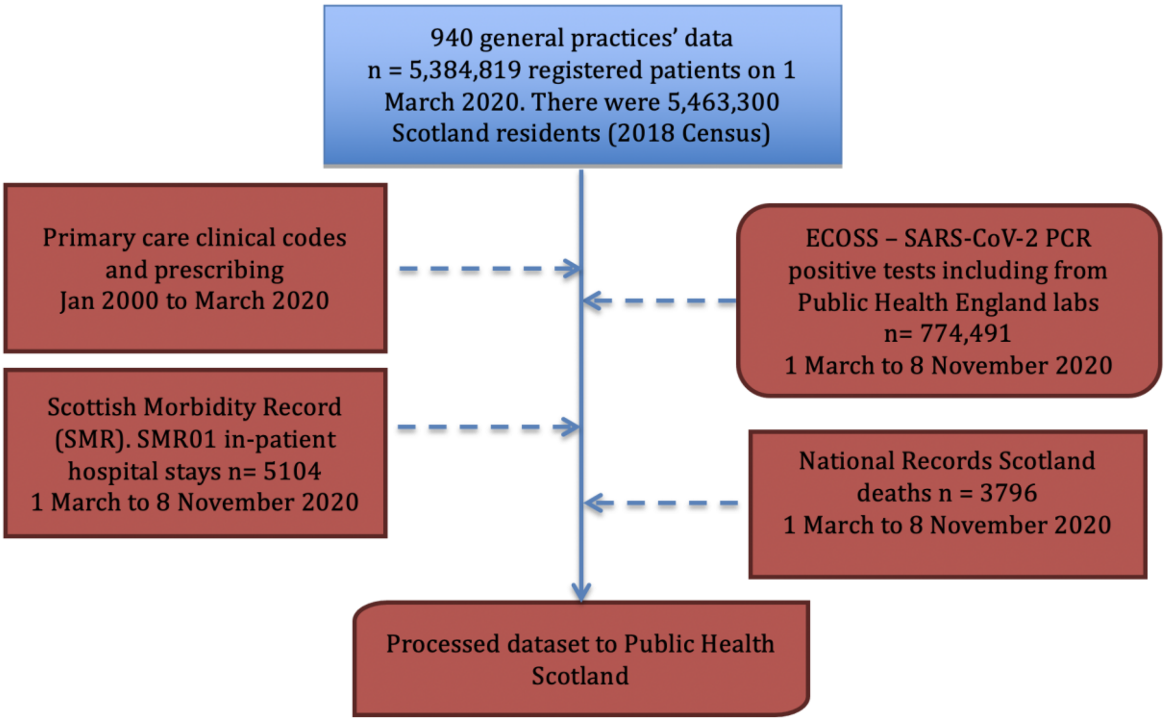

Supplementary Figure 2 Weekly total tests performed throughout the study period

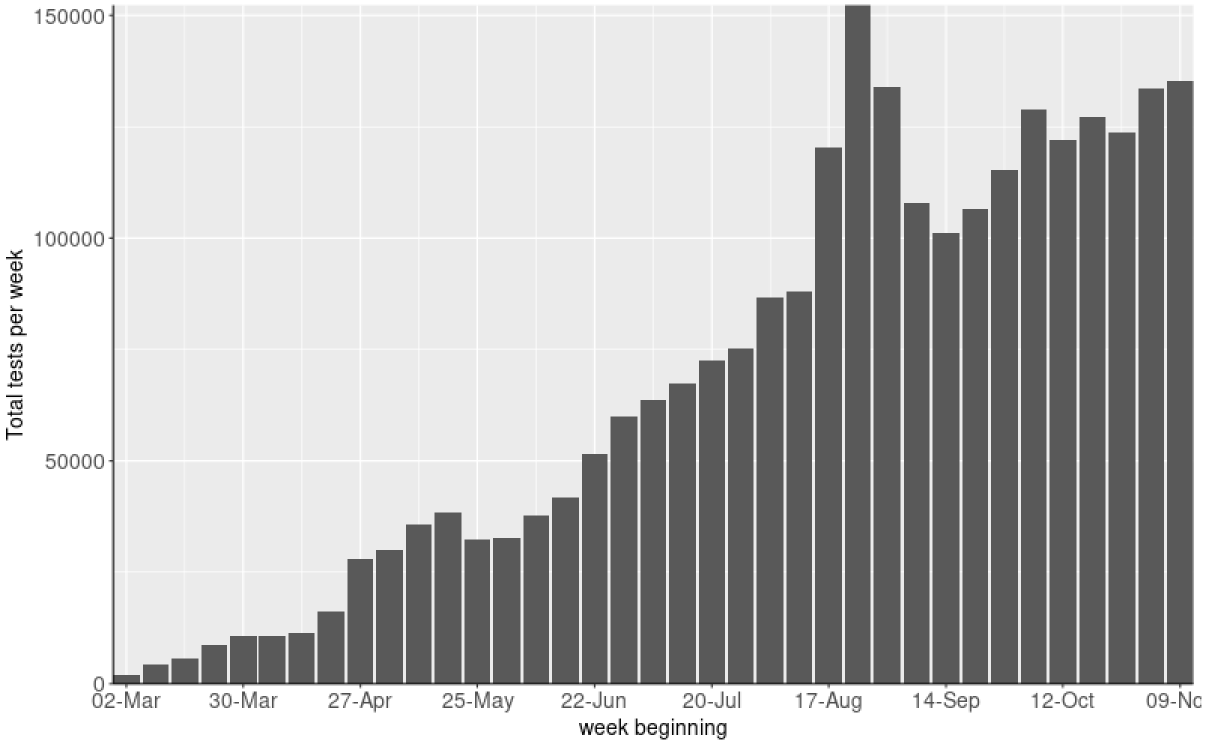

Data from: https://www.opendata.nhs.scot/dataset/covid-19-in-scotland/resource/2dd8534b-0a6f-4744-9253-9565d62f96c2. Schools reopened 11 August 2020. Please note that individuals may have been tested more than once in a week."

Supplemental Figure 3: RT-PCR confirmed SARS-CoV-2 cases over time for females

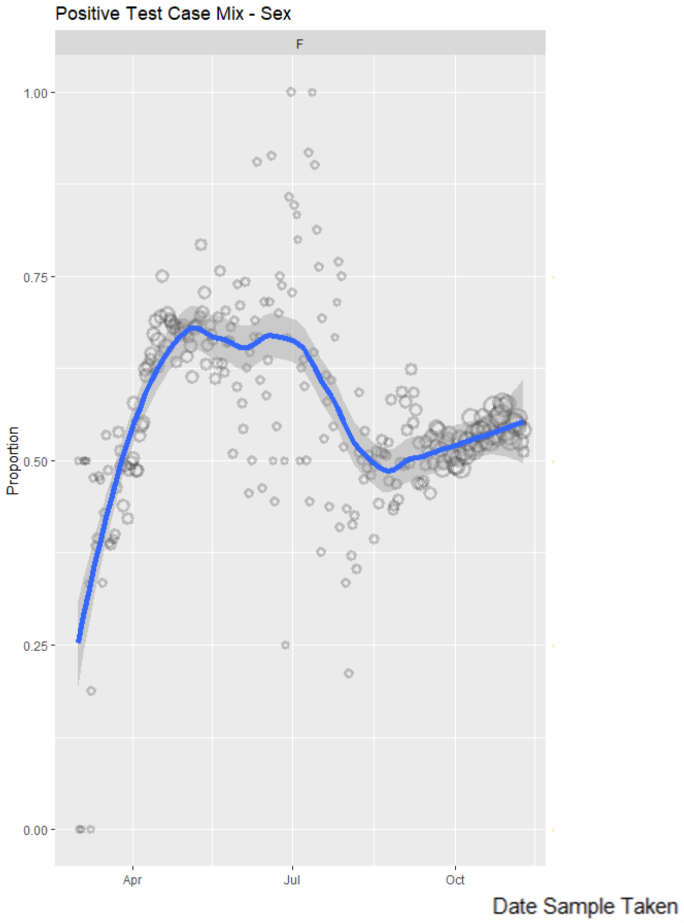

Using data from 75,173 RT-PCR confirmed cases to 8 November 2020 for all people resident with general practices (n=5,384,819). Each dot represents the proportion of participants tested positive on a given day who are female (vs. male). Over the period July and August 2020 there were few positive tests per day and so the proportions are quite variable then. A smooth locally weighted line is plotted in blue (loess) to show the changing temporal trend in the gender distribution among those testing positive.

Supplemental Figure 4: RT-PCR confirmed SARS-CoV-2 cases over time by age band

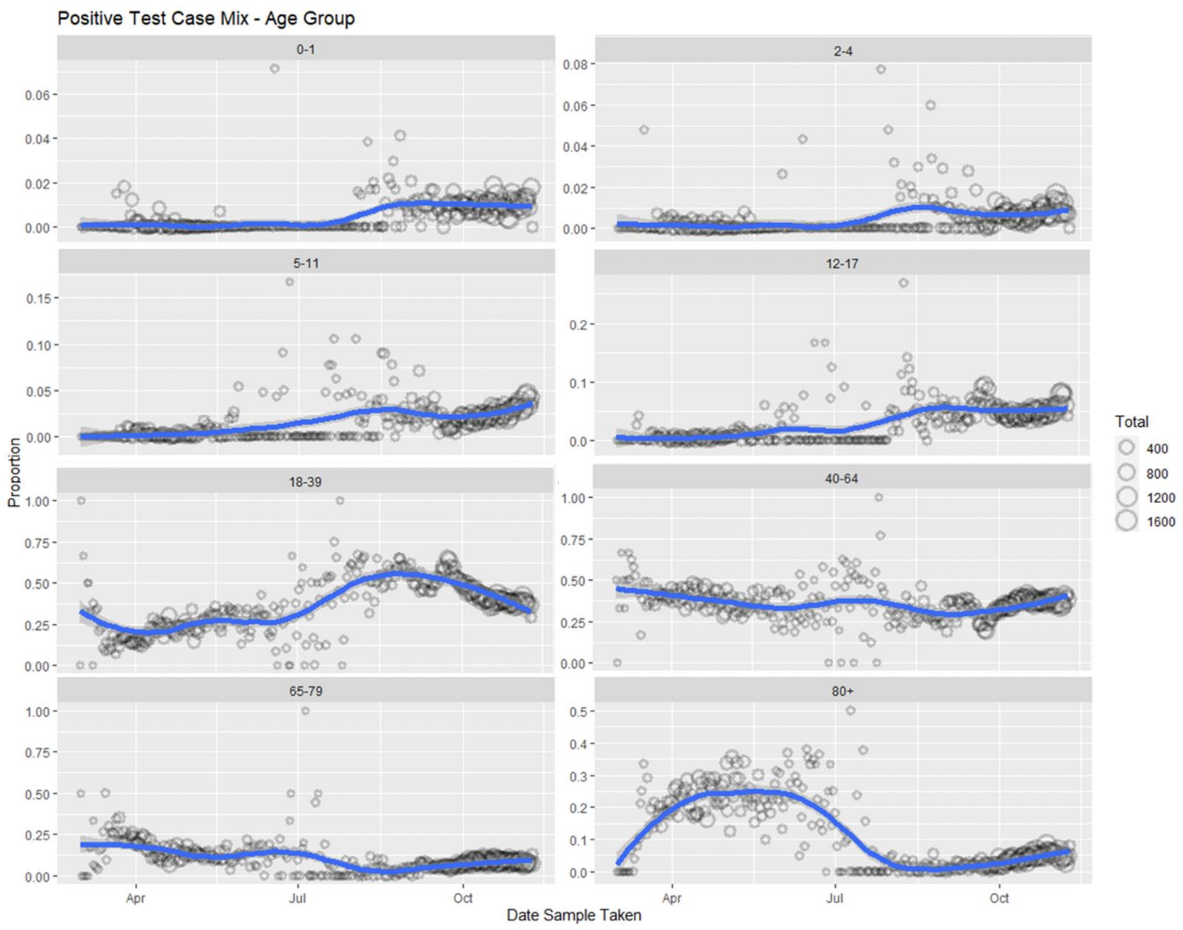

Using data from 75,173 RT-PCR confirmed cases to 8 November 2020 for all people resident with general practices (n=5,384,819). Each dot represents the proportion of participants tested positive on a given day who are in each age group. These proportions add up to one each day. Over the period July and August 2020 there were few positive tests per day and so the proportions are quite variable then. A smooth locally weighted line is platted in blue (loess) to show the changing temporal trend in the age distribution among those testing positive.

Supplemental Figure 5 RT-PCR confirmed SARS-CoV-2 cases over time for risk groups (co-morbidities) and Urban/Rural settings

A

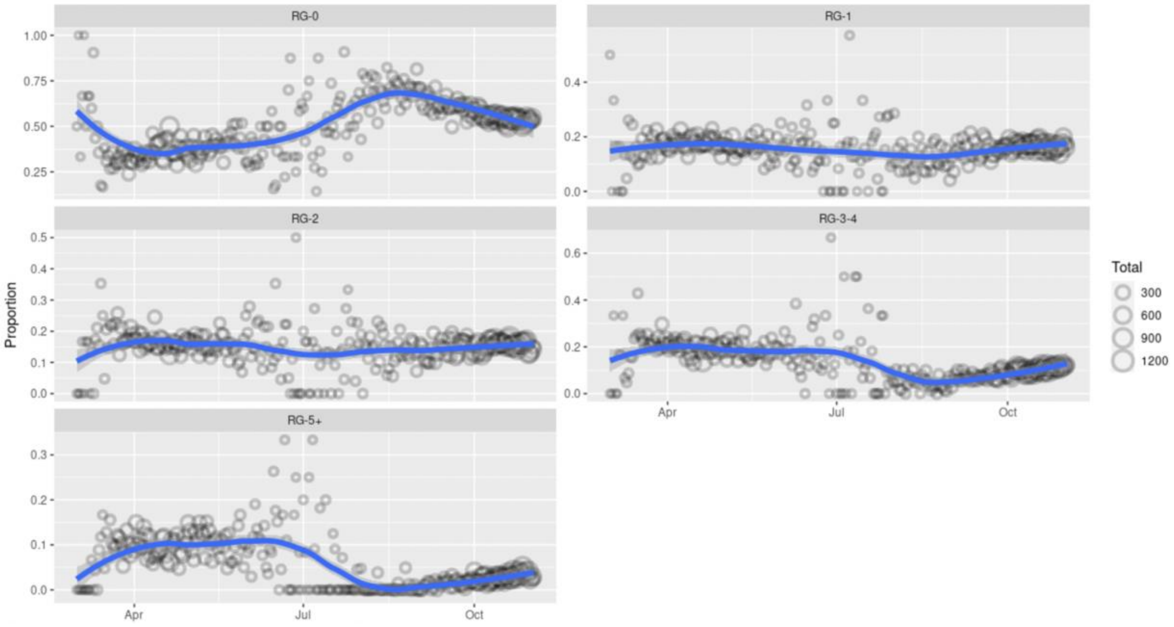

B

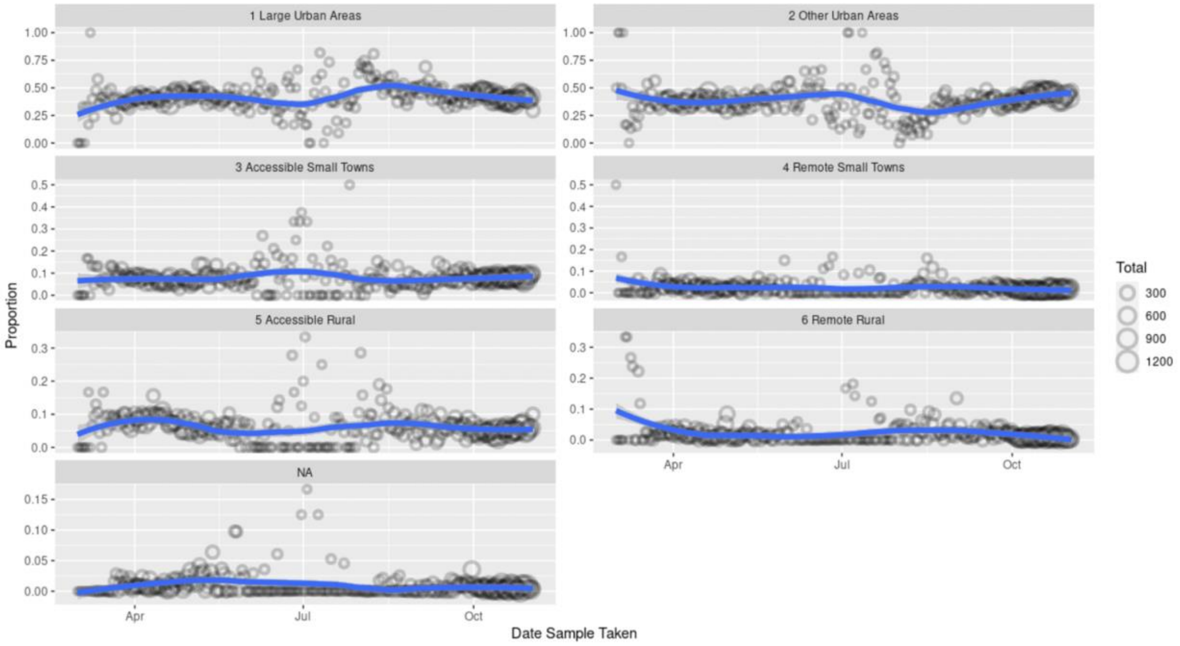

RG - Risk Group. Each dot represents the proportion of participants tested positive on a given day who are in each co-morbidity Risk Group (A) or Urban/Rural location (B). These proportions add up to one each day. Over the period July and August there were few positive tests per day and so the proportions are quite variable then. A smooth locally weighted line is platted in blue (loess) to show the changing temporal trend in the age distribution among those testing positive

Supplemental Figure 6 RT-PCR confirmed SARS-CoV-2 cases over time for socioeconomic status quintiles

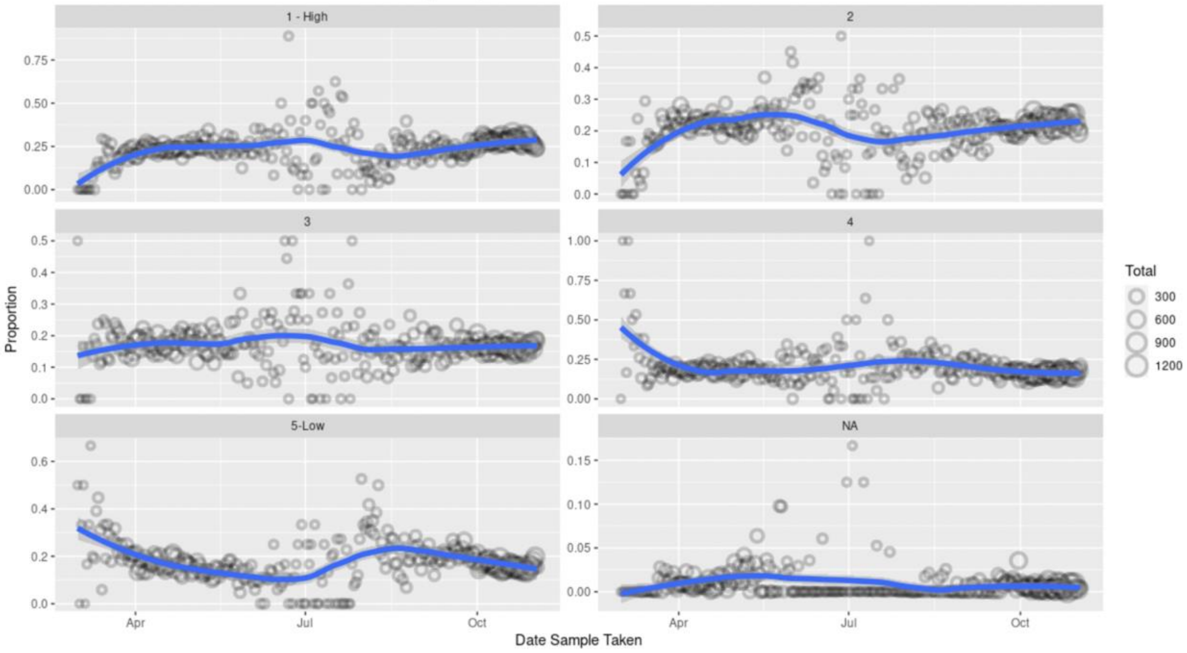

Each dot represents the proportion of participants tested positive on a given day who are in each quintile of socioeconomic status. These proportions add up to one each day. Over the period July and August there were few positive tests per day and so the proportions are quite variable then. A smooth locally weighted line is platted in blue (loess) to show the changing temporal trend in the age distribution among those testing positive

Supplemental Figure 7 Hospitalisation and death forecasts on October 24th 2020

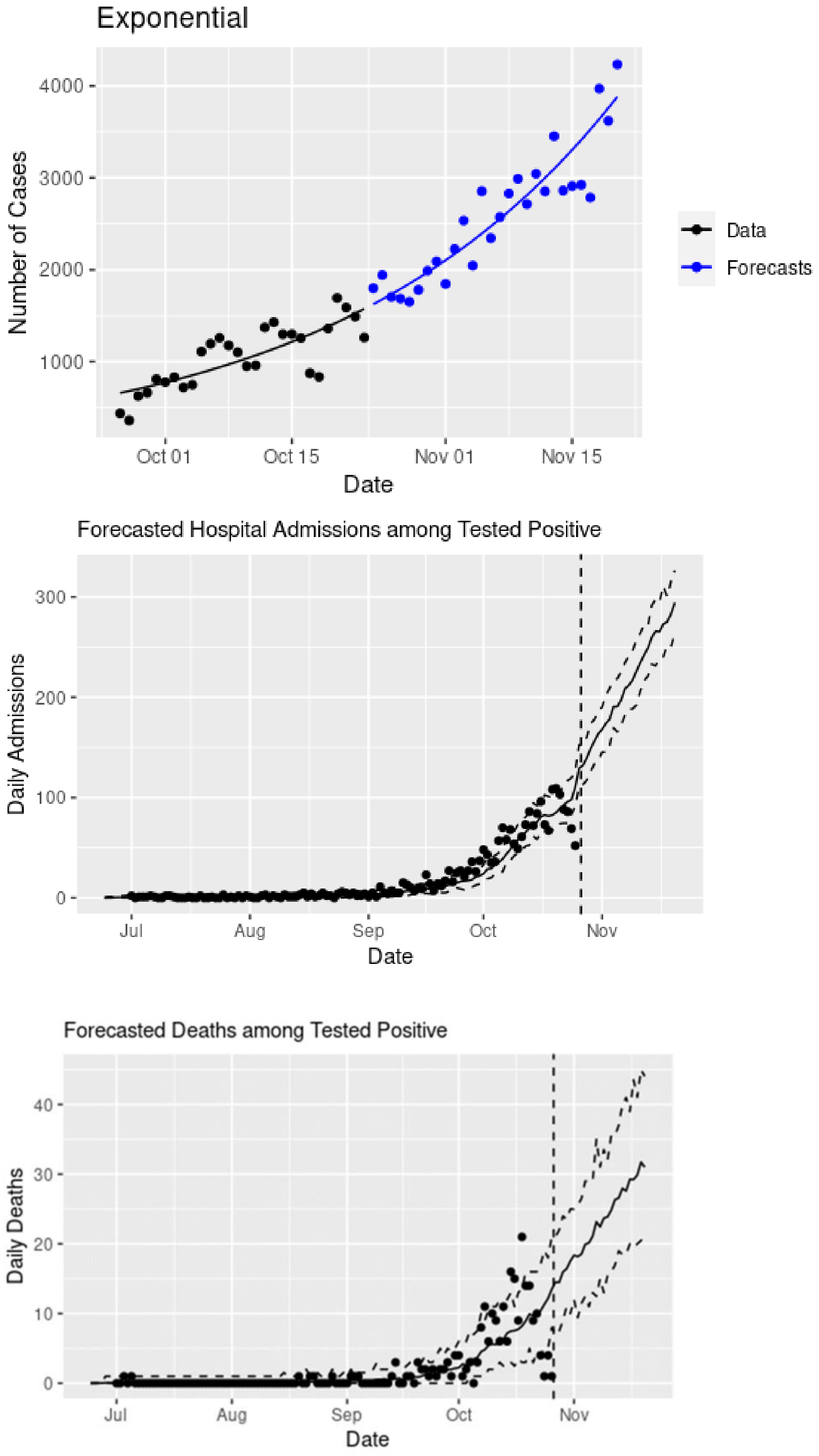

Supplemental Figure 8 Hospitalisation and death forecasts on October 24th 2020 assuming that the growth rate is halved

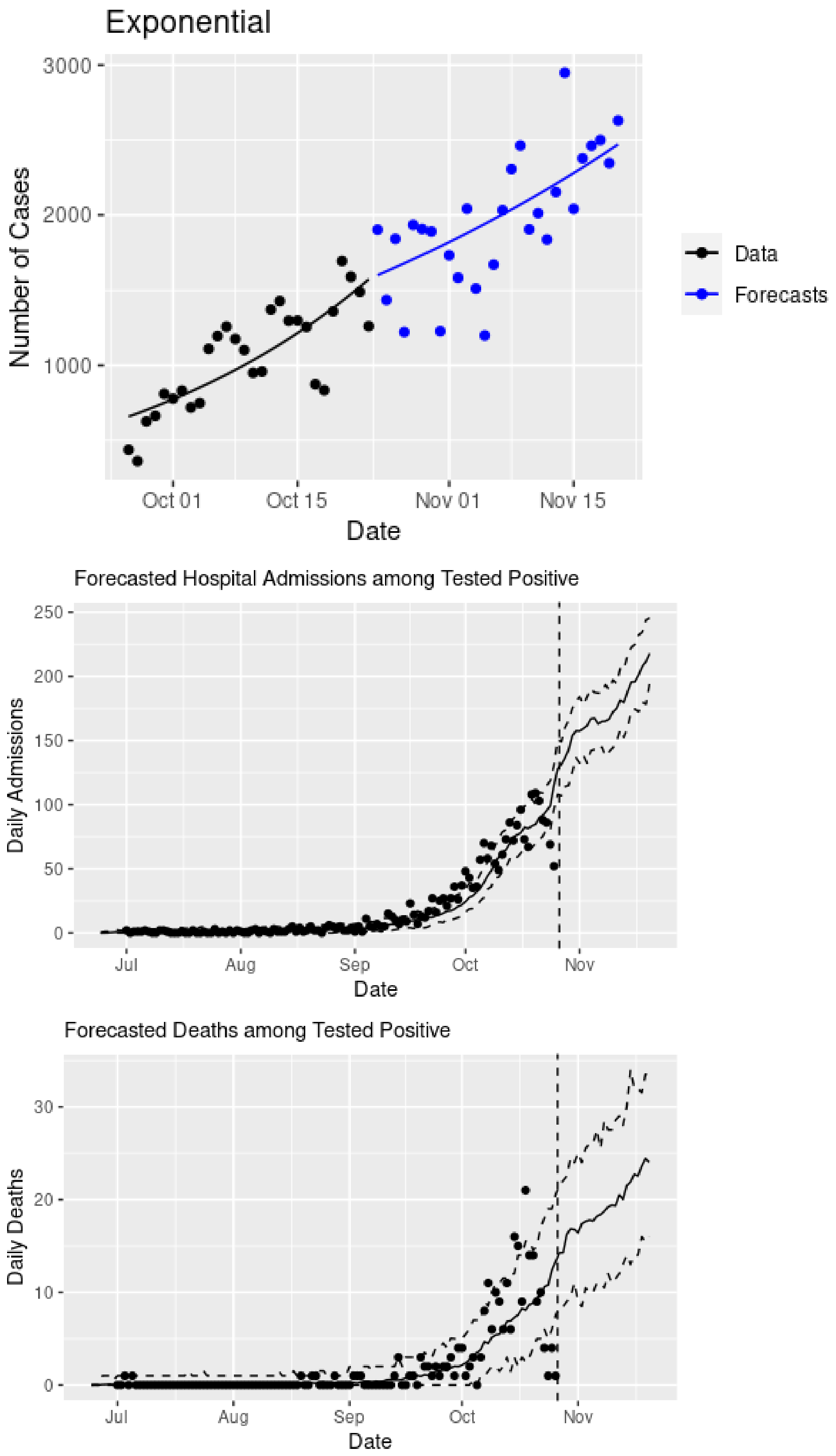

Supplemental Figure 9 Hospitalisation and death forecasts on October 24th 2020 assuming that the growth rate is zero

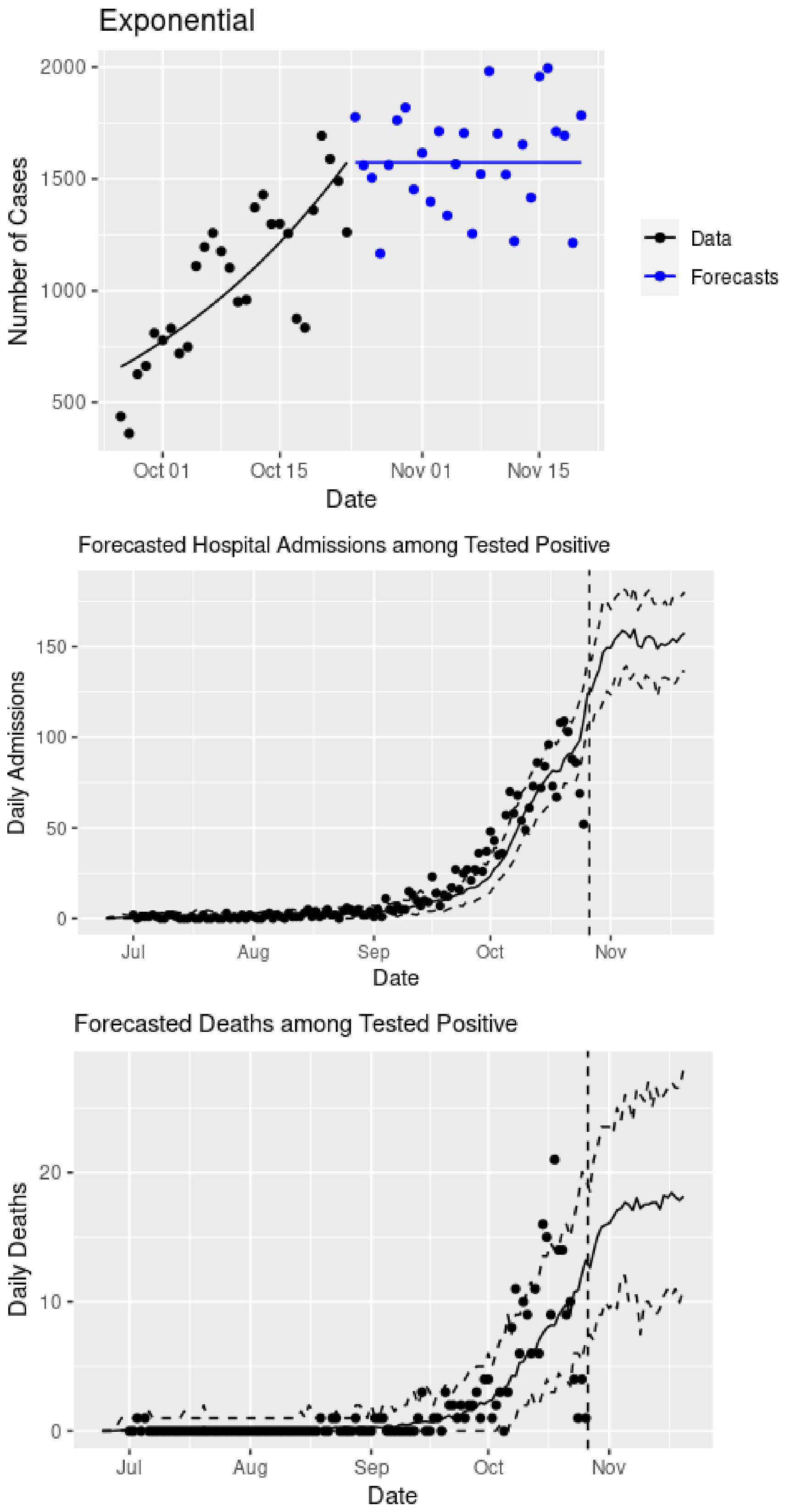

References

1. Health Protection Scotland. Data and surveillance. 2020. https://www.hps.scot.nhs.uk/data/ (accessed 20 July 2020).

2. Simpson CR, Lone NI, Kavanagh K, et al. Evaluating the effectiveness, impact and safety of live attenuated and seasonal inactivated influenza vaccination: protocol for the Seasonal Influenza Vaccination Effectiveness II (SIVE II) study. *BMJ Open* 2017; **7**(2): e014200.

3. National Services Scotland. Information services division. data quality assurance. 2020. https://www.isdscotland.org/Products-and-Services/Data-Quality/Assessments/index.asp?Co=Y (accessed 18.05.2020).
